# Case report: desensitization of hypersensitivity against the antisense oligonucleotide volanesorsen

**DOI:** 10.3389/falgy.2023.1201807

**Published:** 2023-06-08

**Authors:** Rafael H. Isaac, Deyanira Gonzalez-Devia, Carlos O. Mendivil, Edgardo Chapman

**Affiliations:** ^1^ Department of Internal Medicine, School of Medicine, Universidad de El Bosque, Bogotá, Colombia; ^2^ Section of Endocrinology, Department of Internal Medicine, Fundación Santa Fe de Bogotá, Bogotá, Colombia; ^3^ School of Medicine, Universidad de los Andes, Bogotá, Colombia

**Keywords:** triglycerides, familial chylomicronemia syndrome, pancreatitis, volanesorsen, adverse reactions, urticaria

## Abstract

Familial chylomicronemia syndrome (FCS) is a rare autosomal recessive metabolic disorder that causes extremely elevated plasma triglyceride levels, with limited therapeutic options. Volanesorsen is an antisense oligonucleotide approved for its treatment. A 24-year-old woman with genetically diagnosed FCS secondary to a pathogenic variant in *APOA5* and a history of recurrent hypertriglyceridemia-induced pancreatitis episodes was being treated with volanesorsen, 285 mg every 2 weeks. Treatment with volanesorsen achieved normalization of triglycerides to <200 mg/dl. However, after the fifth dose of the medication, the patient developed urticaria and volanesorsen was discontinued. In the absence of alternative pharmacological treatments, the patient received a novel desensitization protocol for volanesorsen that allowed continuation of therapy, without evidence of hypersensitivity reactions after subsequent administrations. FCS requires aggressive multimodal therapy and close follow-up. Volanesorsen has shown great efficacy, but a significant rate of discontinuation due to side effects has been observed. Here, the patient presented an immediate hypersensitivity reaction to volanesorsen, but the provision of a desensitization protocol was effective, facilitating continued treatment and impacting the survival and quality of life of the patient.

## 1. Introduction

Familial chylomicronemia syndrome (FCS) is a rare autosomal recessive disorder with an estimated prevalence of 1 in 1,000,000. The clinical presentation can occur in childhood or early adulthood, and no sex preference has been noted. The major characteristics of FCS are severe hypertriglyceridemia (HTG) with values usually exceeding 10 mmol/L (885 mg/dl) (1) and persistent or intermittent fasting chylomicronemia. While not all patients present clinical signs of the disease, some may present incidental HTG, gallstones, bloating, asthenia, hiporexia, *lipemia retinalis*, eruptive xanthomata, abdominal pain, or acute pancreatitis. FCS develops as a result of a pathogenic single nucleotide or copy-number variant point in genes encoding the enzyme lipoprotein lipase or its regulators (APOC2, APOA5, GPIHBP1, and LMF1). In contrast to FCS, multifactorial hypertriglyceridemia with levels of 500–880 mg/dl is associated with polygenic forms involving multiple simultaneous variants (2).

FCS severely compromises the quality of life, with symptoms ranging from difficulty in concentrating and “brain-fog” to fear of a new episode of abdominal pain or pancreatitis, feeling powerless about the disease, impairment of the ability to fulfill responsibilities at work or school, and an associated absenteeism of 30 days per year on average (3).

Options for long-term management are limited and consist mainly of a severe restriction in total dietary fat intake to <10%–15% of daily calories (15–20 g per day). Other therapies include omega-3 fatty acids, niacin, high-dose fibrates, and statins, with limited and variable responses and a persistent risk of recurring acute pancreatitis (4). Inhibition of the secretion of apolipoprotein C-III (apoC-III) increases the clearance of triglyceride-rich lipoproteins, including chylomicrons and VLDL. Volanesorsen (Waylivra) (ISIS 304801; ISIS-ApoC-III Rx) is a second-generation 20-nucleotide 2′-O-methoxyethyl chimeric antisense oligonucleotide (ASO) that selectively binds the 3′untranslated region of the *APOC3* messenger ribonucleic acid (at base position 489–508), inhibiting its translation and inducing its degradation. This results in 70%–90% lower-circulating apoC-III levels, decreases in plasma triglycerides of between 56% and 86%, improved insulin resistance, reduced rates of acute pancreatitis, and improved overall quality of life (5, 6).

Volanesorsen is administered subcutaneously and metabolized via endonuclease hydrolysis. It is bound to plasma proteins by more than 97%, with a half-life that approaches 14 days, a rapid distribution phase (*t*_max_ 2–4 h), and a slower elimination phase. Steady-state plasma concentrations are achieved approximately at week 13.

Cessation of volanesorsen due to adverse events occurs in up to 30% of patients. The most frequent adverse events are thrombocytopenia (33%–76%) and injection-site reactions (61%), and may include nausea, weakness, myalgia, arthralgia, diarrhea, epistaxis, hyperglycemia, abdominal pain, nasopharyngitis, fatigue, headache, serum sickness, proteinuria, and cholangitis (5, 6). Antibodies against volanesorsen were observed in preclinical trials (16%–30%); nonetheless, they were not associated with reduced drug efficacy or adverse effects. Hypersensitivity to volanesorsen has not been previously reported, and hence, no desensitization protocol has been established (5, 6).

## 2. Case presentation

The patient was a 24-year-old woman with a 3-year history of recurrent hypertriglyceridemia-induced pancreatitis, well-controlled hypothyroidism in which autoimmunity was ruled out, and an isolated self-reported episode of allergic rhino-conjunctivitis to dust mites in 2014. The first episode of acute pancreatitis occurred with serum triglycerides of 3,260 mg/dl, a second episode with 4,876 mg/dl, and the last episode with 11,578 mg/dl. She had no eruptive xanthoma, corneal arcus, hepatosplenomegaly, neurological alterations, and lipodystrophy, and she was not overweight. She was not diagnosed with diabetes nor did she have a family history of dyslipidemia. She did not report consuming alcohol or medications other than levothyroxine, gemfibrozil, glargine insulin (as a lipoprotein lipase activator), and an omega-3 fatty acid supplement. She had no evidence of biliary obstruction or hypercalcemia. Inpatient therapy consisted of bowel rest, intravenous fluids, analgesia, insulin infusion, and fibrates. At discharge, therapy included insulin, fibrates, statins, omega-3 free fatty acids, exercise, and dietary restrictions with a low-fat/carbohydrate diet. Her triglycerides oscillated between 300 and 5,000 mg/dl depending on dietary adherence. Genetic tests were performed, identifying a loss-of-function heterozygous mutation in exon 3 of the gene *APOA5* [c.(50-1)_(161 + 1_162-1)]. The gene *APOAV* encodes apolipoprotein A-V, a cofactor that stabilizes the dimers of lipoprotein lipase and is essential for its enzymatic activity at the endothelium.

**Figure 1:**
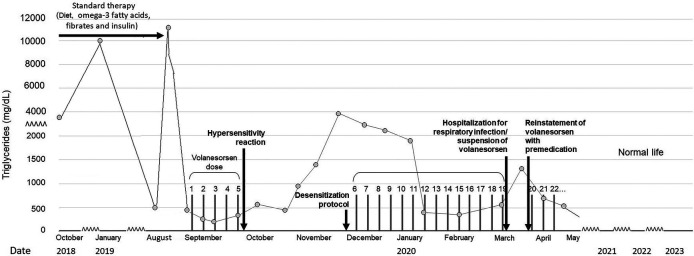
Evolution of plasma triglycerides before and after the volanesorsen desensitization protocol.

**Figure 2:**
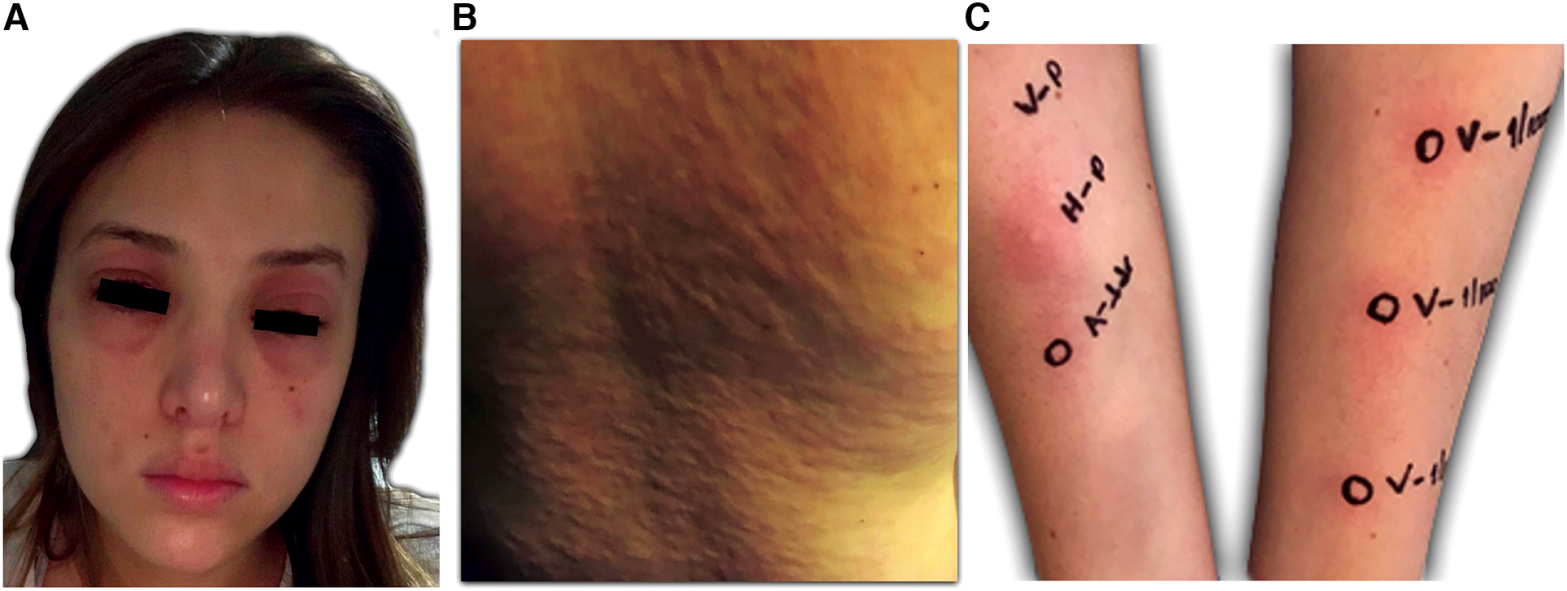
Clinical presentation of the patient. (**A**) and (**B**) Itchy welts, nettle rash, and angioedema 20 min after the application of volanesorsen. (**C**) Confirmation of hypersensitivity to volanesorsen. Volanesorsen 285 mg/1.5 ml (190 mg/ml), prick test was performed with the undiluted solution and intradermal skin tests 1/100 (1.9 mg/ml) and 1/10 (19 mg/ml). In both dilutions, the increase in the initial papule was more than 20% of its size and there was a halo of associated erythema.

**Figure 3:**
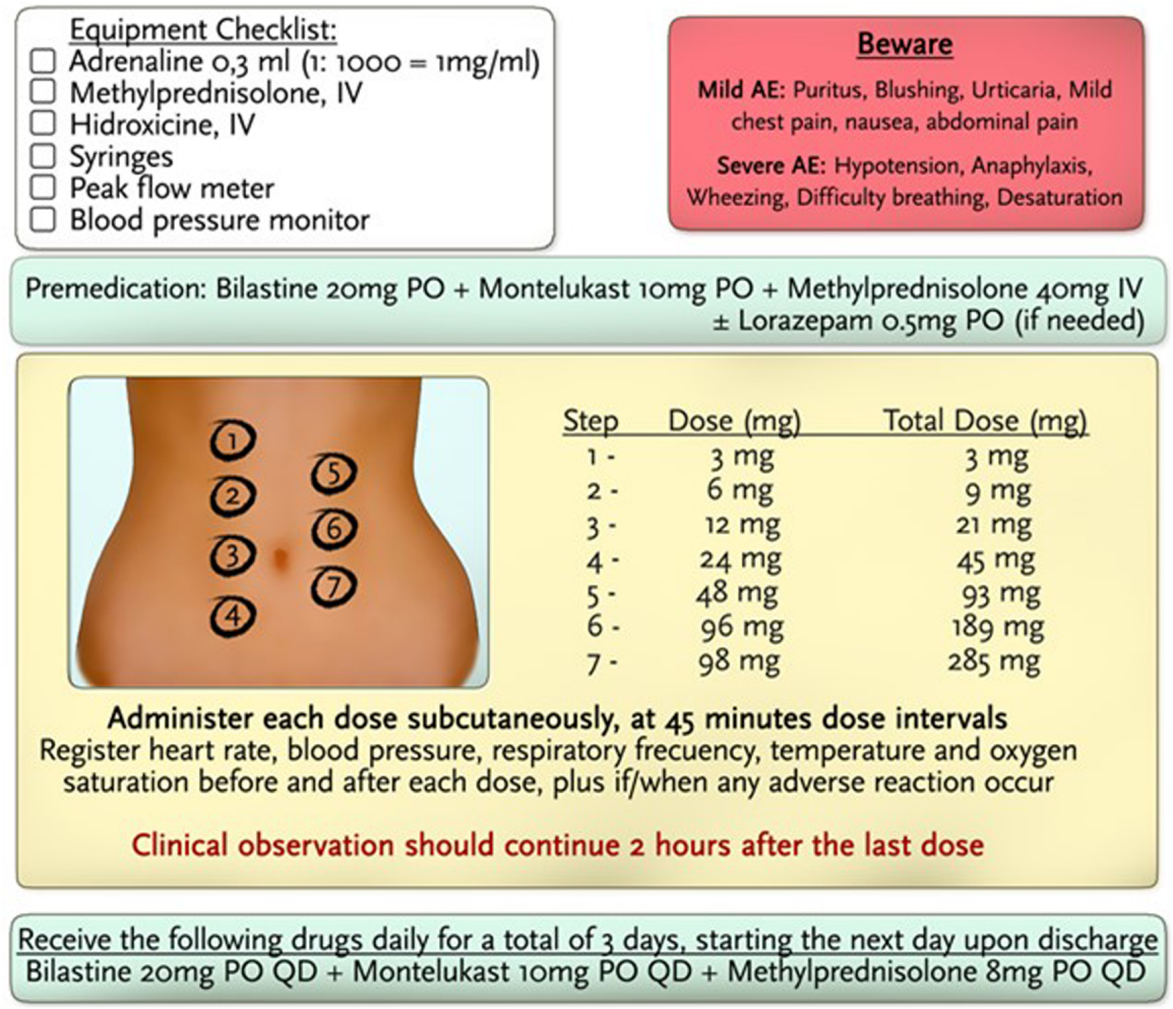
Desensitization protocol for volanesorsen.

Despite all pharmacologic and non-pharmacologic interventions, the patient was refractory to standard therapy, and volanesorsen with a dosage of 285 mg SC to be taken every week was started. During the course of the month, the patient received volanesorsen, and she sustained normal serum triglyceride levels (<200 mg/dl, Figure 1). At the fifth dose, 20 min after administration, the patient developed facial angioedema, accompanied by evanescent, severely pruriginous, erythematous, and papular lesions localized in the torso, face, and arms, compatible with urticaria (Figure 2, panels A–C). Tryptase serum levels were obtained at 7.4 μg/L (normal range 0–11), and serum serotonin was 6.2 ng/ml (normal range 56–246); antibodies against volanesorsen were negative. Hypersensitivity to volanesorsen was causally confirmed by using Naranjo's scale, WHO-UMC, and VCAT. Therapy with volanesorsen was discontinued and treatment with montelukast, methylprednisolone, and clemastine was required to control symptoms, which slowly abated over 4 days. There was no relationship between the presence of symptoms and acute infection and the intake of non-steroidal anti-inflammatory drugs or alcohol. Over the 8 weeks following the cessation of volanesorsen, plasma triglyceride levels increased steadily until they reached 2,500 mg/dl, a level that represents a stage of critical risk for acute pancreatitis (Figure 1). Considering that no protocols for desensitization to volanesorsen were established, and no effective pharmacological alternatives existed for this patient, a desensitization protocol was developed in conjunction with the Brigham and Women's Hospital—Harvard Medical School Workforce led by Dr. M. Castells (Section 3, and Figure 3). Thus, 9 weeks after the discontinuation of volanesorsen, the patient provided informed consent and was admitted to the intensive care unit to undergo this desensitization protocol.

The patient continued to receive volanesorsen weekly for 3 months after desensitization with good control of plasma TG (Figure 1), and was then admitted again to the hospital with generalized myalgia, arthralgia, chills, malaise, and fainting with functional limitation, 6 h after the application of volanesorsen (in an upper limb). She did not report a cough, dyspnea, wheals, skin or mucous membrane lesions, or clinical symptoms of urticaria, angioedema, or anaphylaxis. Her vital signs were heart rate 87 bpm, breathing rate 18, temperature 36.2°C, and oxygen saturation 94% in room air. Her laboratory results showed leucocytes 14,000/mm^3^, neutrophils 93.2%, lymphocytes 2.9%, hemoglobin 12.9 g/L, hematocrit 34.3%, platelet count 224,000/mm^3^, erythrocyte sedimentation rate 3 mm/h, creatinine 0.87 mg/dl, glucose 114 mg/dl, creatine phosphokinase 54 UI/L, sodium 133 mmol/L, potassium 3.49 mmol/L, chloride 103 mmol/L, total calcium 9 mg/dl, phosphorus 3.3 mg/dl, magnesium 1.69 mg/dl, negative rheumatoid factor normal, triglycerides 916 mg/dl, C-reactive protein 2,643 mg/dl, anticyclic citrullinated peptide antibodies 41 UI/ml, serum tryptase 4.73 mcg/L, and negative antinuclear antibodies. SARS-Cov-2 infection was ruled out. The clinical diagnosis was an acute respiratory infection, unrelated to the prior episode of hypersensitivity to volanesorsen, but the agent had been temporarily suspended during her hospitalization.

After hospitalization, the clinical care team discussed the option of restarting volanesorsen with prior use of a day of nonsteroidal anti-inflammatory drugs such as naproxen 500 mg every 12 h and montelukast 10 mg prior to the procedure and then 72 h of surveillance and close communication with the healthcare staff. This conclusion was reached after discussing the risk/benefit ratio of continuing volanesorsen considering the lack of a feasible alternative in Colombia, the risk of serious complications and death associated with pancreatitis, as well as the thrombotic risk associated with hypertriglyceridemia.

The patient has now completed 36 months of follow-up with regular application of volanesorsen with premedication, presenting good control of triglycerides, improved quality of life, and without any other significant adverse event. She feels very satisfied with the normalization of her plasma TG and the avoidance of any further episodes of pancreatitis. This has allowed her to take up a job and start her professional career without the extreme dietary restrictions, constant gastrointestinal symptoms, and sense of impending pancreatitis risk that she had to endure prior to her treatment with volanesorsen.

## 3. Desensitization protocol

Premedication 1 hour prior to the protocol consisted of bilastine 20 mg PO, methylprednisolone 40 mg IV, and montelukast 10 mg PO. The administration of volanesorsen was performed in 7 steps in 45-min dose intervals. Dilutions were used until the total dose of 285 mg was completed, and the total dose was administered in 5.25 h. The amount of drug delivered in each subsequent step was approximately twice the dose of the previous step. No adverse events were noted during the administration of the protocol. Subsequently, quantities of full-dose volanesorsen were administered in a hospital setting with careful monitoring of vital signs, blood glucose, and systemic allergic reactions. No evidence of hypersensitivity reactions or any other adverse events has been recorded thus far.

## 4. Discussion

The goal of the desensitization protocol was to administer suboptimal doses, to make the mast cells and basophils unresponsive to antigens either by administering an excess of monomeric antigens that cannot bind to IgE-specific receptors or by the rapid internalization of binding receptors from the cell membrane, without affecting the function of the mastocytes or basophils when faced with other stimuli.

Drug desensitization is a therapeutic procedure that allows an individual allergic to a drug to be able to temporarily tolerate it, in the knowledge that the drug is essential and cannot be replaced by another for the treatment of the underlying disease. Desensitization is achieved through the consecutive administration of small doses of the culprit drug until the full therapeutic dose is reached. The goal of the procedure is to administer suboptimal doses to the patient that will promote small stimulation of mast cells/basophils, inducing inhibitory mechanisms and rendering these cells hyporesponsive (7, 8).

In the case of this patient, it was necessary to desensitize her to volanesorsen because it was the only effective treatment available for her, and because there was a history of adverse effects and therapeutic failures in the attempt to reduce her plasma triglyceride levels with other strategies.

## 5. Conclusion

Treatment of FCS is challenging, and most current therapies are only marginally effective in preventing extreme hypertriglyceridemia and pancreatitis. Volanesorsen has shown great efficacy but has a significant rate of discontinuation due to side effects. We present a desensitization protocol that offers an alternative to patients with hypersensitivity to volanesorsen, in whom no other options currently exist to prevent HTG and acute recurrent pancreatitis. This offers the possibility of improving their quality of life and eventually their overall chances of survival.

## Data Availability

The original contributions presented in the study are included in the article, further inquiries can be directed to the corresponding author.
